# Enhancement of the Seed Color, Antioxidant Properties, and Agronomic Traits of Colored Wheat via Gamma Radiation Mutagenesis

**DOI:** 10.3390/foods14030487

**Published:** 2025-02-03

**Authors:** Min Jeong Hong, Chan Seop Ko, Jin-Baek Kim, Dae Yeon Kim

**Affiliations:** 1 Advanced Radiation Technology Institute, Korea Atomic Energy Research Institute, 29 Geumgu, Jeongeup 56212, Republic of Korea; hongmj@kaeri.re.kr (M.J.H.); csko@kaeri.re.kr (C.S.K.); jbkim74@kaeri.re.kr (J.-B.K.); 2 Department of Plant Resources, College of Industrial Sciences, Kongju National University, 54 Daehak-ro, Yesan-eup, Yesan-gun 32439, Republic of Korea

**Keywords:** wheat, gamma radiation, seed color, diversity, antioxidant activity

## Abstract

Wheat, a staple crop cultivated for over 8000 years, sustains more than 2.5 billion people globally, as a major source of carbohydrate, protein, fiber, and essential nutrients. Colored wheat, enriched with dietary fiber and antioxidants, offers valuable genetic resources for developing functional wheat varieties. Herein, a mutant pool of 1069 colored wheat lines was developed through gamma-ray irradiation to enhance genetic diversity. Mutant lines were classified into 10 groups based on seed color parameters (L*, a*, and b*), which were measured using the Hunter Lab system. *K*-means clustering categorized the mutant lines, and four representative lines from each group were analyzed for agronomic traits (plant height, spike length, thousand-seed weight, and kernels per spike) and antioxidant properties (radical-scavenging activity, ferric reducing antioxidant power, and total antioxidant capacity). Principal-component analysis revealed distinct clustering patterns, indicating associations between seed color, agronomic traits, and antioxidant activity. Darker seed color groups exhibited 3–16% higher levels of bioactive compounds and 10–18% higher antioxidant activities, whereas lighter groups showed 8–42% lower functional potential compared to the control wheat. These findings highlight the potential of mutation breeding in generating phenotypic diversity and developing wheat varieties with improved functional traits and bioactive compound content.

## 1. Introduction

Wheat is one of the first domestic crops and has been cultivated for >8000 years, with approximately 220 million hectares currently under cultivation [1]. It is one of the most important cereal crops, consumed by >2.5 billion people worldwide as a staple food. It is a major source of complex carbohydrates and provides important nutrients, including fiber and protein, as well as minor constituents such as vitamins, minerals, and phytochemicals [2]. Wheat is commonly milled into flour and used to prepare food products, such as bread, cookies, noodles, and pasta. The demand for wheat is rising as it serves both as a staple food and a source material for various products.

In wheat breeding programs, breeders aim to increase grain or biomass yield, enhance resistance or tolerance to biotic and abiotic stresses, and improve end-use quality. The decrease in cultivated area due to climate change is a major cause of wheat production loss and deterioration in quality. Various studies are being conducted to increase wheat yield and maintain quality through the development of resistant crops. It is important to not only secure enough food but also obtain good-quality food for human health. Enhanced production of wheat containing many functional substances has attracted increasing attention, and several studies have been conducted [3,4,5].

Colored wheat (CW) is an optimal breeding material that contains many nutrients and dietary fiber. Seed color is associated with the properties and nutritional and antioxidant capacities of the seed, which determine its commercial value. Wheat exhibits various seed colors, such as white, red, green, blue, purple, and black [6,7,8]. In recent years, new goals such as biofortification have emerged to meet consumer demand for a healthy diet. Commonly cultivated wheat is white- or red-grained; however, considerable attention has been paid to the development of CW varieties. CW is characterized by enhanced free-radical-scavenging capacity and high anthocyanin content [9,10]. The seed color of CW is categorized as purple, blue, and black, depending on the type and location of anthocyanins in the wheat seed layer [11,12]. Purple pigmentation is observed in the pericarp layer, and blue pigmentation is caused by the presence of anthocyanins in the aleurone layer of the wheat grain [13]. In black wheat grain, anthocyanin is present both in the pericarp and aleurone layer [14]. Anthocyanin is one of the representative antioxidants and helps prevent various diseases, such as inflammation, obesity, diabetes, cardiovascular diseases, cancer, and aging [15]. Consistent intake of wheat grain anthocyanins can be an effective approach to promote health.

CW contains considerably higher levels of health-promoting compounds, such as anthocyanins and phenolic compounds, than common wheat (white- or red-grained wheat) [16]. These bioactive compounds, particularly anthocyanins, are largely absent in common wheat, making CW nutritionally superior in terms of antioxidant properties [17]. Traditional breeding for CW encounters various challenges, such as limited genetic resources, narrow phenotypic diversity, and the time-intensive nature of the process, making it difficult to simultaneously improve yield, uniformity, and nutritional traits [3]. Developing populations with broader genetic diversity facilitates the selection of plants with novel characteristics and offers a means of overcoming these limitations. Despite its nutritional benefits, CW remains underutilized because of issues related to large-scale cultivation, including poor uniformity and lower yields. Nonetheless, its potential as a breeding material for enhancing nutritional quality and functional traits makes it a valuable option for plant breeders. Mutation breeding has emerged as a robust method for broadening plant genetic diversity and overcoming the limitations of conventional approaches [18]. This enables the development of unique traits, such as novel seed colors and improved functional properties, thereby increasing the agronomic and nutritional value of CW. Among various mutation-inducing techniques, gamma-radiation-induced mutagenesis is one of the most effective and widely used methods in modern breeding programs. By introducing random genetic mutations, gamma irradiation facilitates the development of novel phenotypic traits that are difficult to achieve through conventional breeding. Of the mutant varieties registered in the Mutant Variety Database, 63% of the varieties developed using physical mutagens have employed gamma irradiation, underscoring the critical role of gamma irradiation in facilitating genetic innovation [19]. Calrose 76—a representative rice variety developed via gamma irradiation—was cultivated in California until the late 1970s; it remains a notable example of the practical application of gamma irradiation in crop improvement [20]. Since its development, gamma-radiation-induced mutagenesis has been extensively employed to enhance wheat’s key agronomic traits, including seed weight, yield, and grain quality. Previous studies have demonstrated the effectiveness of this method in improving productivity and resilience across various crops [21,22,23].

CW has garnered attention for its enriched bioactive compounds, such as anthocyanins and phenolic compounds, which offer potential health benefits [10]. However, the genetic and phenotypic variability within CW populations remains underexplored, and the relationship between seed color and functional traits, such as antioxidant capacity, is not fully understood. While previous studies have highlighted the nutritional advantages of CW [4,8,10], there is limited information on how its diverse pigmentation correlates with both agronomic performance and phytochemical content.

In this study, a mutant pool of CW lines was constructed using gamma irradiation to address these knowledge gaps and broaden the genetic diversity available for breeding high-performance CW. A total of 1069 CW mutant lines were developed and classified according to seed color. Several variants representing each group were selected and used for analyzing agronomic traits, phytochemical content, and antioxidant capacity. This research proposes that seed color serves as a phenotypic marker for bioactive compound content and antioxidant activity in CW mutants, offering insight into the potential of mutation breeding for developing nutritionally superior wheat varieties.

## 2. Materials and Methods

### 2.1. Plant Materials and Agronomic Traits

CW served as the parental variety for developing a gamma-radiation-induced mutant population. CW is characterized by deep-purple seeds, a plant height of approximately 75 cm, and low winter hardiness. In total, 200 g of CW seeds were gamma-irradiated at the Korea Atomic Energy Research Institute (KAERI, Jeongeup, Republic of Korea) using a ^60^Co gamma irradiator (150 TBq capacity; Nordion, ON, Canada) at a dose rate of 25 Gy/h, delivering a total dose of 200 Gy. Following irradiation, the CW seeds were sown in rows in a radiation breeding field. M_1_ plants with distinct traits were selected, and their spikes were harvested. The seeds were then propagated for subsequent generations. From M_2_ to M_6_, the mutant lines exhibiting diverse phenotypes, such as plant height, spike shape, seed color, and flowering time, were selected. Through continuous selection, 1069 stable mutant lines derived from CW were obtained in the M_7_ generation_._ These lines were categorized into 10 groups based on seed color, with 4 lines randomly selected from each group. In total, 40 CW mutants, along with CW (the original variety used for gamma irradiation) and ‘Keumkang’, were used for subsequent experiments. Keumkang, which is Korea’s most widely cultivated white wheat variety, was included as a reference to evaluate how the mutant lines perform against a high-yielding, commercially important wheat variety. Wheat seeds were sown and cultivated in a radiation breeding field (35.5699° N and 126.9722° E) (Jeongeup-si, Jeollabuk-do, Republic of Korea) during the 2022–2023 growing season. Agronomic traits were measured to evaluate yield potential and adaptability, focusing on key indicators critical for wheat improvement. Days to heading (HD) was recorded when >50% of the wheat spikes of each mutant line had emerged. Plant height (PH) was measured from the ground level to the apex of the spike, excluding the awn length. Spike length (SL) was measured as the distance between the base of the first spikelet and the topmost spikelet, excluding the awn length. For thousand-seed weight (TSW), the average weight of 100 seeds in each line was counted, and the weight was multiplied by 10. The number of kernels per spike (NKS) was counted from the main spikes that were randomly selected from each line. These traits collectively provide a comprehensive assessment of the agronomic performance of the mutant lines, contributing to their potential suitability for large-scale cultivation.

### 2.2. Colorimetric Measurement and K-Means Clustering

**Figure 1:**
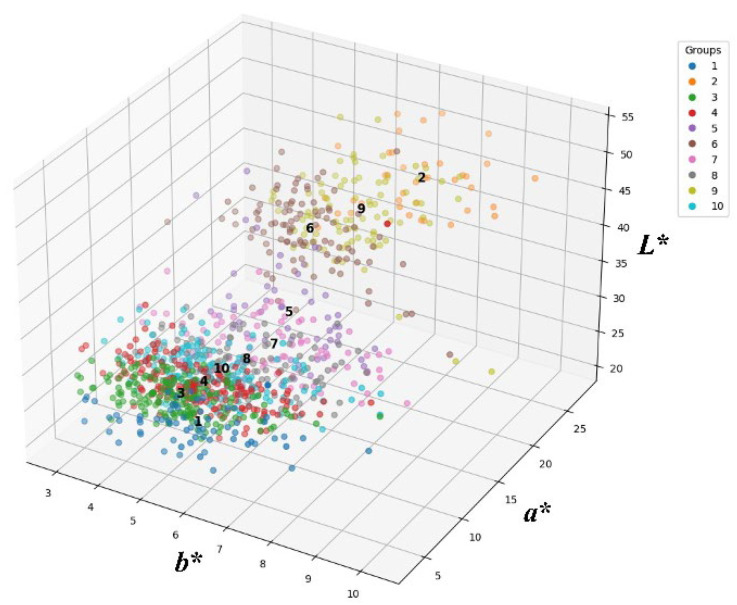
Hunter L*, a*, and b* color diagram for seed color in 1069 mutant lines of colored wheat. A three-dimensional scatter plot of L*, a*, and b* values for seed color in 1069 colored wheat mutant lines, grouped into 10 clusters using *K*-means clustering. Each point represents a mutant line, and colors indicate assigned clusters. Axes L*, a*, and b* correspond to lightness, redness–greenness, and yellowness–blueness, respectively.

Wheat grain color was determined from the Hunter L* (lightness–darkness), a* (redness–greenness), and b* (yellowness–blueness) values, using a colorimeter from the Lab model Colormate (Scinco, Korea). Black and white tiles were used to calibrate the instrument with a D65 illuminant. Each grain sample was placed in a dedicated container before recording the color parameters. Based on the Hunter color values (L*, a*, and b*), which were measured using ColorMaster software 2017 version (Scinco, Seoul, Republic of Korea), *K*-means clustering was performed to classify 1069 CW mutants into 10 groups. Clustering analysis was conducted using MeV 4.9.0 software, with the number of clusters (k) predefined as 10 [24]. This grouping served as the basis for downstream analyses, including the characterization of seed color variability, agronomic traits, and antioxidant properties. The clustering results are presented in a three-dimensional scatter plot (Figure 1), showing the distribution of L*, a*, and b* values across the 10 groups. Violin plots were constructed to visualize the distribution of L*, a*, and b* values within each cluster. Quartiles and density distributions were indicated inside the violin plots, and the mean values of the reference groups, including the wild types, CW, and Keumkang, were overlaid as horizontal dashed lines.

### 2.3. Determination of Total Anthocyanin Content

Total anthocyanin content was determined using the spectrophotometric method described by Mita et al. [25]. A 0.5 g homogenized wheat sample was weighed, mixed with 10 mL of methanol–HCl (1% HCl, *w*/*v*), and incubated at 4 °C for 24 h. All samples were centrifuged at 14,000 rpm for 20 min, after which the supernatant was collected and filtered through a 0.2 μm filter. The absorbance of the supernatant was measured at 530 and 657 nm using a UV–Vis spectrophotometer (Evolution 260 Bio, Thermo Scientific, Waltham, MA, USA). The anthocyanin content was calculated using the following formula: Q = (A530 − 0.25A657) × M − 1, where Q indicates the concentration of total anthocyanin, A530 and A657 represent absorption at 530 and 657 nm, respectively, and M represents the weight of homogenized grain.

### 2.4. Seed Sample Preparation

Approximately 1 g of seeds from each line was finely ground using a homogenizer and extracted with 80% methanol (10 mL) via sonication for 30 min in an ultrasonic bath at 25 °C. The seed extracts were filtered to remove debris using a 0.45 μm filter and stored at −20 °C until use. The prepared seed extracts were used to measure antioxidant capacity (2.5), total phenolic compound content (2.6), and flavonoid content (2.7).

### 2.5. Antioxidant Capacity Assay

The 2,2-diphenyl-1-picrylhydrazyl (DPPH) assay was performed according to the method of Brand-Williams et al. [26]. In total, 0.2 mL of seed extract was mixed with 3.8 mL of DPPH solution. The mixture was incubated at room temperature for 30 min under dark conditions. Absorbance was immediately measured at 517 nm using a UV–Vis spectrophotometer (Thermo). The radical-scavenging capacity of each sample was calculated as follows: DPPH scavenging activity = (A_control_ − A_sample_/A_control_) × 100, where A_sample_ represents the absorbance of the reaction mixture with seed extracts, and A_control_ indicates the absorbance value of the reaction mixture without extracts.

The 2,2′-azino-bis (3-ethylbenzothiazoline-6-sulfonic acid) diammonium salt (ABTS) assay was performed using the method of Re et al. [27]. In total, 7 mM ABTS ammonium and 2.45 mM potassium persulfate were mixed, and the mixture was incubated for 12 h at room temperature under dark conditions for ABTS radical production. The mixture was diluted with methanol (80%) until the absorbance at 734 nm reached 0.7, as measured using a spectrophotometer. Then, 10 μL of seed extract was mixed with 190 μL of ABTS, and the reaction mixture was incubated at room temperature for 6 min. The color change was assessed by measuring absorbance using a spectrophotometer plate reader at 734 nm. ABTS radical-scavenging capacity was calculated as follows: ABTS scavenging activity = (A_control_ − A_sample-_/A_control_) × 100, where A_control_ indicates the absorbance of the control (blank), and A_sample_ indicates the absorbance of the seed extract.

The ferric reducing antioxidant power (FRAP) assay was performed according to the manufacturer’s protocol using a FRAP assay kit (Sigma, Saint Louis, MO, USA). Absorbance was measured at 595 nm in the kinetic mode for 60 min at 37 °C using a spectrophotometer plate reader (Bio-Rad, Hercules, CA, USA). FRAP values, expressed in mM ferrous equivalents of the samples, were derived from the standard curve.

The total antioxidant activity (TAC) of wheat seeds was determined using a commercial kit (MAK187, Sigma, Saint Louis, MO, USA), according to the manufacturer’s instructions. Antioxidant activity was measured by detecting reduced Cu^+^ ion chelate at a wavelength of 570 nm using a colorimetric probe. In total, 10 μL of each sample was diluted with an equal volume of 50-fold-diluted Cu^2+^ reagent. The samples were mixed and incubated for 90 min at room temperature under dark conditions. The absorbance of each sample was measured at 570 nm using a spectrophotometer plate reader. Absorbance was calculated based on the value obtained from “Trolox equivalents” using a standard curve.

### 2.6. Measurement of Total Phenolic Compounds

The phenolic compound content was determined using a commercially available phenolic compound assay kit (Sigma), according to the manufacturer’s protocol. In total, 40 μL of seed extract (up to 100 μL with distilled water), 20 μL of PC probe, and 80 μL of PC assay buffer were mixed and incubated at room temperature for 10 min under gentle shaking. Absorbance was recorded at 480 nm using a spectrophotometer plate reader (Bio-Rad, Hercules, CA, USA) in the end-point mode. The concentration of phenolic compounds was calculated using a catechin standard curve.

### 2.7. Measurement of Flavonoid Content

The total flavonoid content in the ethanol extracts of wheat seeds was quantified using a plant flavonoid colorimetric assay kit (Elabscience, Houston, TX, USA). Homogenized wheat seeds (0.02 g) were extracted with 60% (*v*/*v*) ethanol at 60 °C for 2 h. After centrifugation, the supernatant was used for the assay. The reaction mixture was prepared according to the manufacturer’s protocol. Absorbance was measured against a blank at 510 nm using a spectrophotometer plate reader (Bio-Rad). The total flavonoid content was calculated by plotting the optical density values of the standard on the *y*-axis and the corresponding concentration on the *x*-axis.

### 2.8. Principal-Component Analysis and Statistical Analysis

Principal-component analysis (PCA) was performed to reduce dimensionality and identify clustering patterns across 1069 CW mutant lines. The analysis incorporated seed color traits (L*, a*, and b*), biochemical traits (anthocyanins, phenolic compounds, flavonoids, and proanthocyanidins), antioxidant activities (DPPH, ABTS, FRAP, and TAC), and agronomic traits (PH, SL, HD, TSW, and NKS). All data were normalized to *z*-scores prior to analysis to ensure comparability across scales. PCA was conducted using Python (version 3.12) with the Scikit-learn library, and the results were visualized using plotlib and seaborne. Scatter plots and loading plots were generated to illustrate the clustering of mutant lines and the contribution of individual traits to the principal components. One-way analysis of variance was used to evaluate differences in seed color traits (L*, a*, and b*), biochemical traits, antioxidant activities, and agronomic traits across the 10 groups classified by *K*-means clustering. Duncan’s multiple-range test was employed as post hoc analysis to identify pairwise differences among the groups at a significance level of *p* < 0.05. Statistical analyses were conducted using Python (version 3.12) with the scipy and statsmodels libraries. Visualizations, including boxplots, violin plots, and correlation matrices, were generated using plotlib and seaborne to highlight the differences between the groups and correlations between traits.

## 3. Results

### 3.1. Classification of CW Mutants Based on Seed Color

**Figure 2:**
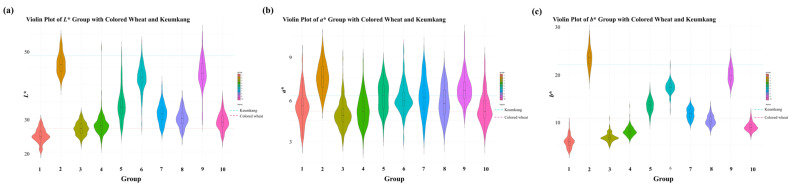
Violin plots displaying the distribution of (**a**) L*, (**b**) a*, and (**c**) b* values across 1069 colored wheat mutants, grouped into 10 clusters based on *K*-means clustering. Each cluster represents a distinct group identified through the clustering of L*, a*, and b* values. The quartiles are marked within the violin plots, and the horizontal dashed lines indicate the L*, a* and b* values for the wild types, colored wheat, and Keumkang.

CW seeds were irradiated with 200 Gy of gamma rays and then sown in rows in a radiation breeding field. Distinct M_1_ plants were selected based on phenotypic traits, and their spikes were harvested for subsequent propagation. From M_2_ to M_6_, mutant lines displaying diverse phenotypes, including PH, spike shape, seed color, and flowering time, were identified through continuous field screening. By the M_7_ generation, 1069 stable mutant lines with a range of agronomic and morphological traits were established. These mutant lines were classified into 10 groups based on seed color. The classification was conducted by measuring three color parameters (L*, a*, and b*) using the Hunter Lab color system. A wide diversity in seed color values was observed among the CW mutants. The Hunter L*, a*, and b* color diagram for seed color in 1069 CW mutant lines is presented in Figure 1. Based on L*, a*, and b* values, the mutants were classified into 10 groups using *K*-means clustering. The number of mutants belonging to each group and the mean L*, a*, and b* values in each group are shown in Table S1. The average L*, a*, and b* values for the seed colors of 1069 CW mutants were 33.62, 5.96, and 13.36, respectively (Table 1). The value ranges of L*, a*, and b* were 20.26–53.31, 2.83–10.29, and 3.39–27.68, respectively (Figure 2). Among the 10 groups, Groups 1 and 2 exhibited the largest difference. a* exhibited limited variation, leading to minimal differences between the groups; however, L* and b* demonstrated clear differences among the groups (Table 1). Among the 10 groups, Groups 3 and 4, which exhibited seed colors similar to the wild type (CW, L*: 27.024, a*: 5.1597, and b*: 10.6111), harbored the greatest number of CW mutant lines (189 and 183 lines, respectively). In contrast, Group 2, in which seed color was absent, contained the fewest (only 39) mutant lines. Groups 2 and 9 displayed L* and b* values comparable to those of Keumkang (L*: 48.520; a*: 6.1885; b*: 21.7579). Group 1, which had the darkest seed color, comprised 67 lines. The L* value of this group was lower than that of CW, indicating reduced lightness. Moreover, the b* value of this group was significantly lower than that of CW, reflecting a more intense hue. These results confirm that Group 1 exhibited a seed color that was substantially darker than that of the wild type (CW), emphasizing its distinct phenotypic difference compared with other groups. After gamma irradiation, a selection of wheat lines allowed for the observation of a diverse range of seed colors distinct from that of the wild type.

### 3.2. Variations in Active Compounds and Antioxidant Activities by Seed Color

**Figure 3:**
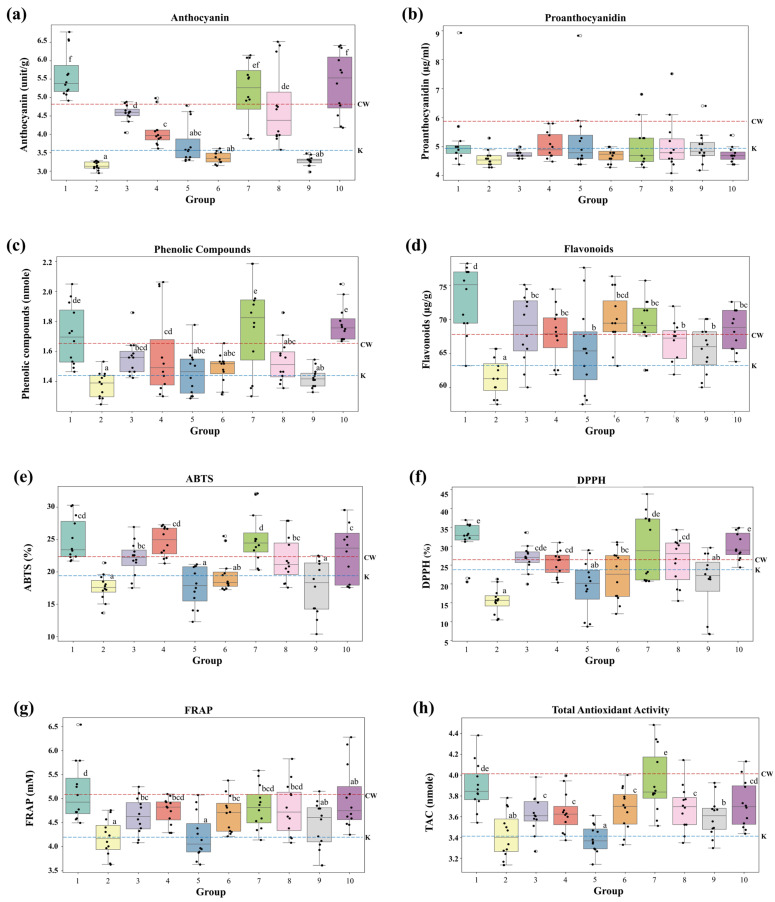
Variation in the contents of anthocyanins, proanthocyanidins, phenolic compounds, and flavonoids as well as the levels of antioxidant activities of the colored wheat mutant according to seed color. (**a**) Anthocyanin content, (**b**) proanthocyanidin content, (**c**) phenolic compound content, (**d**) flavonoid content, (**e**) ABTS radical-scavenging activity, (**f**) DPPH radical-scavenging activity, (**g**) FRAP radical-scavenging activity, and (**h**) total antioxidant capacity (TAC). Each box plot represents the distribution of each trait across the clusters. The central line in each box plot represents the median value, and the whiskers denote the range of the data. Statistical comparisons were performed using analysis of variance followed by Duncan’s multiple-range test (*p* < 0.05). Different letters above the boxes indicate statistically significant differences between the groups. The red and blue dotted lines in each figure represent the values of CW and Keumkang (K), respectively.

Figure 3 shows the distribution of anthocyanins, proanthocyanidins, phenolic compounds, and flavonoids as well as the antioxidant activities measured by ABTS, DPPH, FRAP, and TAC across each group. Except for proanthocyanidins, the levels of the abovementioned compounds and antioxidant activities differed significantly (*p* < 0.05) among the groups with different seed colors (Figure 3 and Table 2). Anthocyanin content was the highest in Groups 1 (5.6195 units) and 10 (5.375 units), whereas Groups 2 (3.147 units), 5 (3.747 units), and 9 (3.275 units), in which the seed color had faded, exhibited relatively lower anthocyanin levels (Figure 3a). Groups 1, 7, and 9 exhibited higher anthocyanin content than the control (CW). Despite variations in seed color, proanthocyanidin levels did not differ significantly among the groups (Figure 3b). This could be attributed to the fact that proanthocyanidins primarily contribute to red pigments, whereas other pigments, such as anthocyanins, which dominate in purple wheat, account for the color differences without significantly affecting proanthocyanidin content [28]. The phenolic compound content was higher in Group 10 (1.786 nmole) than in CW (1.650 nmole), with Groups 7 (1.768 nmole) and 1 (1.708 nmole) also showing relatively elevated levels (Figure 3c). Flavonoid content was the highest in Group 1 (73.10 μg/g) and lowest in Group 2 (61.50 μg/g), whereas CW displayed a flavonoid content of 67.25 μg/g (Figure 3d). ABTS radical-scavenging activities were higher in Groups 1 (24.974%), 4 (24.692%), and 7 (25.179%), all of which exceeded that of CW (Figure 3e). Groups 1 (31.659%), 7 (29.874%), and 10 (30.067%) had the highest DPPH activity, whereas Group 2 (15.537%) displayed the lowest activity (Figure 3f). Notably, Groups 1 and 10 showed higher DPPH activity than CW. FRAP radical-scavenging capacity was the highest in Group 1 (5.133%) and lower in Groups 2 (4.180%) and 5 (4.212%) (Figure 3g). TAC exhibited the highest antioxidant activity in Groups 1 (3.893 nmole) and 7 (3.935 nmole) and lowest activity in Group 5 (3.388 nmole) (Figure 3h). Based on these results, it can be inferred that Group 1, with a darker seed color, exhibited higher levels of active compounds and antioxidant activity, whereas Groups 2 and 9, which lost their color characteristics and displayed lighter seed colors, showed relatively lower activity.

### 3.3. Correlation Analysis and PCA

**Figure 4:**
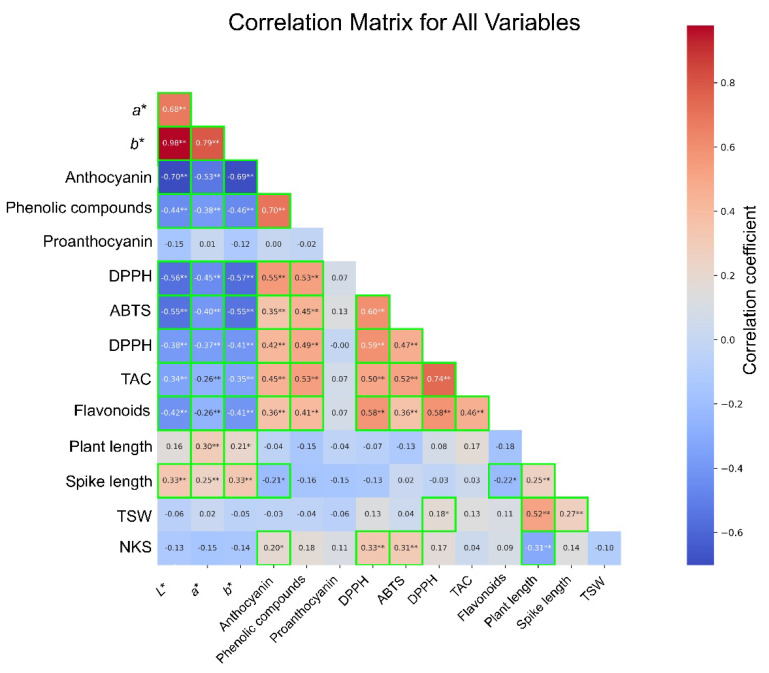
Correlation matrix illustrating the relationship among agronomic traits, seed color parameter (L*, a*, and b*), anthocyanins, proanthocyanidins, phenolic compounds, flavonoids, and antioxidant activities of the colored wheat mutants. Each cell in the matrix displays the Pearson correlation coefficient between two variables, with the strength and direction of the correlation indicated by the value. Correlations were considered significant at *p* < 0.05 (*) and *p* < 0.01 (**). Green boxes highlight statistically significant correlations at *p* < 0.05 or below, emphasizing key interactions between traits.

To evaluate the correlation between seed color, agronomic traits, and antioxidant traits, a correlation analysis of 40 selected samples was conducted, representing 10 distinct groups generated through *K*-means clustering of 1069 CW mutant lines (Figure 4). Pearson’s correlation analysis revealed correlations among the examined parameters, with significance observed at different levels. L*, a*, and b* values, which indicate seed color brightness, exhibited significant negative correlations with anthocyanins (L*: r = −0.70, *p* < 0.01; a*: r = −0.53, *p* < 0.01; and b*: r = −0.69, *p* < 0.01). This suggests that the darker seeds with lower L*, a*, and b* values are associated with higher anthocyanin content. Similarly, phenolic compounds showed negative correlations with L*, a*, and b* values (r = −0.38 to −0.70, *p* < 0.01), and a strong positive correlation was observed with anthocyanins (r = 0.70, *p* < 0.01).

Darker seeds, which are rich in anthocyanin and phenolic compounds, demonstrated higher antioxidant activity. L*, a*, and b* values exhibited negative correlations with antioxidant activities (DPPH, ABTS, FRAP, and TAC), with correlation coefficients ranging from −0.26 to −0.57 (*p* < 0.01). In contrast, anthocyanins and phenolic compounds showed positive correlations with antioxidant activities, with r values of >0.35 (*p* < 0.01). DPPH, ABTS, FRAP, and TAC were significantly and positively correlated with each other (r > 0.47, *p* < 0.01).

Flavonoids showed negative correlations with L*, a*, and b* values (r = −0.42 to −0.36, *p* < 0.05) and positive correlations with anthocyanins (r = 0.58, *p* < 0.01) and phenolic compounds (r = 0.42, *p* < 0.01). Agronomic traits exhibited weaker correlations overall. TSW and PH showed a positive correlation (r = 0.52, *p* < 0.01), whereas SL exhibited weak positive correlations with L*, a*, and b* values (r = 0.33 to 0.38, *p* < 0.05). NKS demonstrated weak positive correlations with anthocyanin, DPPH, and ABTS but showed a negative correlation with PH (r = −0.31, *p* < 0.05). Proanthocyanidins exhibited no significant correlation with any of the parameters.

These results underscore the complex correlations between seed color, bioactive compounds, and antioxidant activities. The darker seeds with higher anthocyanin and phenolic compound contents were associated with enhanced antioxidant activities, whereas the lighter seeds exhibited lower levels of antioxidant activities. Although agronomic traits generally showed weaker correlations with seed color and antioxidant traits, certain parameters, such as TSW and PH, displayed notable correlations, providing insights for further exploration of the trade-offs between agronomic and functional traits.

**Figure 5:**
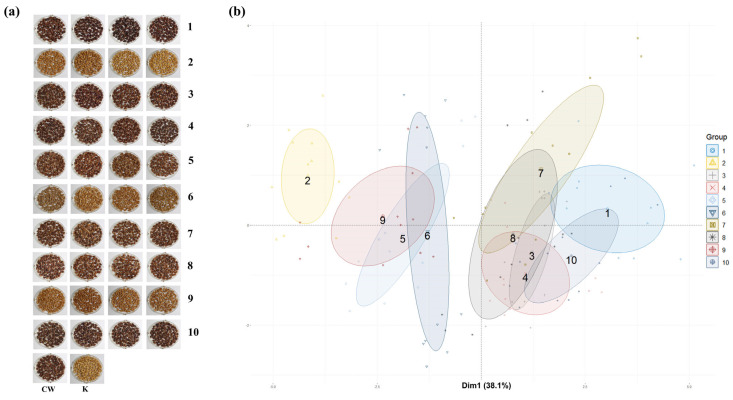
PCA of the experimental data derived from the investigated datasets. (**a**) PCA plot displaying the phenotypic diversity among 10 groups (Groups 1–10), as determined by *K*-means clustering. Each group is represented by four samples, with different colors indicating distinct groups based on seed color and associated phenotypic traits. The bottom row labeled as “CW” shows the control mutant plants, whereas “K” denotes the ‘Keumkang’ variety. (**b**) PCA plot illustrating multivariate correlations among all measured parameters, including seed color indices (L*, a*, and b*), bioactive compounds (anthocyanins, phenolic compounds, proanthocyanidins, and flavonoids), antioxidant activities (DPPH, ABTS, FRAP, and TAC), and agronomic traits (PH, SL, TSW, and number of kernels per spike). Dim1 explains 38.1% of the total variation, indicating it captures the majority of differences in the dataset. Each group is differentiated by unique colors and shapes, displaying clustering patterns and relationships among groups based on the measured traits.

The CW mutant lines developed through gamma irradiation were classified into 10 groups based on their L*, a*, and b* values. Representative seed color images of four randomly selected lines from each group are shown in Figure 5a, illustrating the phenotypic diversity in seed coat color among the groups. PCA was conducted to classify the mutants by integrating L*, a*, and b* values, agronomic traits, anthocyanins, phenolic compounds, proanthocyanidins, flavonoids, and antioxidant activities. As shown in Figure 5b, the scatter plot generated through PCA illustrates multivariate correlations among the groups.

Among the 10 groups, Groups 1 and 2 exhibited the greatest difference, with Group 1 characterized by darker seed colors and Group 2 by lighter, yellowish colors. The distribution patterns revealed that the 10 groups could be broadly divided into two major clusters. Groups 1, 3, 4, 7, 8, and 10 were categorized into one cluster, primarily consisting of darker seed colors with higher anthocyanin levels and antioxidant activities. Groups 2, 5, 6, and 9 formed another cluster, comprising mutants with lighter seed colors, lower levels of active compounds, and reduced antioxidant activities.

This distinction underscores the strong correlation between seed color and functional traits, because darker seeds consistently exhibited higher levels of anthocyanins, phenolic compounds, and antioxidant activities, whereas lighter seeds showed a significant reduction in these parameters. This consistency demonstrates that PCA and *K*-means clustering effectively captured the major variations in seed color, agronomic traits, and antioxidant activities among the mutant lines. The consistency between these methods underscores the robustness of the classification approach used in this study. Specifically, darker seed groups, such as Groups 1 and 10, consistently clustered together in both analyses, reflecting their higher anthocyanin levels and antioxidant activities, whereas lighter seed groups, such as Groups 2 and 9, were consistently positioned separately, corresponding to their lower functional potential. These findings demonstrate that *K*-means clustering, based on L*, a*, and b* values, reliably classifies mutant lines by seed color, and PCA provides further validation by illustrating multivariate correlations with bioactive compound content and agronomic traits.

### 3.4. Variation in Agronomic Traits

**Figure 6:**
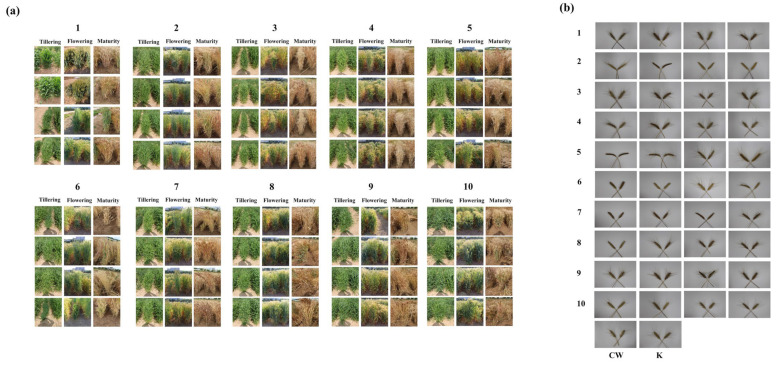
Phenotypes of the 40 selected colored wheat mutant lines. (**a**) Phenotypic changes in plants during the growth period. (**b**) Spike morphology of the colored wheat mutants.

A total of 40 CW mutant lines were used for further experiments, with four mutants that were randomly selected from each group based on L*, a*, and b* values exhibiting representative characteristics (Figure 6 and Table 3). Figure 6a highlights the phenotypic diversity observed during different growth stages (tillering, flowering, and maturity) among the CW mutant lines, whereas Figure 6b illustrates the variations in spike morphology. The mutants demonstrated differences in seed color, which were classified by L*, a*, and b* values, as well as significant variation in agronomic traits. PH ranged from 32.7 to 140.0 cm, whereas SL varied between 6.0 and 13.5 cm. HD also displayed diversity, ranging from 187 to 202. In total, 8/40 mutants exhibited spike morphologies without awns (Figure 6b). TSW ranged from 30.5 to 50.7 g, with a mean value of 40.8 g, whereas NKS varied from 31.3 to 68.7, with a mean value of 47.2.

Although all 40 mutants were selected based on their L*, a*, and b* values, the range of agronomic traits demonstrated significant diversity. Notably, certain mutants outperformed wild-type CW in terms of agronomic traits, such as NKS and TSW. This indicates that gamma irradiation not only induced changes in seed color but also generated mutants with improved agronomic traits. These findings underscore the potential of mutation breeding to enhance functional and agronomic traits, contributing to the development of diverse and superior wheat lines.

## 4. Discussion

Wheat is one of the most widely cultivated and consumed cereal crops globally, serving as a staple food for a substantial proportion of the population [29]. In addition to their nutritional significance, CW varieties have attracted growing interest because of their potential health advantages and distinctive visual characteristics. CW, characterized by pigments in its seed coat, is rich in anthocyanins, flavonoids, and other bioactive compounds with strong antioxidant properties [10]. These compounds not only enhance the nutritional value of wheat but also contribute to reducing the risk of chronic diseases [30]. The diversification of seed color in wheat offers an opportunity to enhance its functional food value and expand its utility in specialty and health-focused food markets. However, traditional breeding methods for achieving diverse seed colors are often time-consuming and limited by genetic variation within the existing germplasm. In this study, gamma-radiation-induced mutagenesis was employed to address these limitations, generating diverse seed coat colors in CW and enabling their classification based on seed color, active compound contents, and antioxidant activity. Furthermore, we investigated the correlation between seed color, active compound content, and antioxidant activity in CW mutants generated through gamma-radiation-induced mutagenesis.

These results demonstrate that seed color is a key determinant of the phytochemical profile of wheat, influencing the levels of anthocyanins, phenolic compounds, flavonoids, and antioxidant activities. Darker seed colors (Group 1) were associated with higher levels of active compounds and greater antioxidant activity, whereas lighter mutants (Groups 2 and 9) showed lower levels of these beneficial compounds. This correlation aligns with the finding of studies suggesting that the presence of anthocyanins and other bioactive compounds contributes to the antioxidant properties of CW [31,32]. The consistency between *K*-means clustering based on seed color and PCA of biochemical and agronomic traits reinforced the consistency of this correlation. The darker groups (Groups 1 and 10) consistently clustered together in both analyses, highlighting their higher bioactive compound content and antioxidant capacities. Conversely, the lighter groups (2 and 9) were positioned separately, reflecting their lower functional potential. This agreement underscores the robustness of seed color as a reliable marker for predicting functional traits. Moreover, the ability to classify mutants based on seed color through *K*-means clustering provides an efficient phenotypic marker for identifying lines with high antioxidant capacity, reducing dependence on labor-intensive biochemical assays during early-stage screening.

The increased antioxidant activity observed in the darker mutants can be attributed to the higher accumulation of anthocyanins, which are strong antioxidants [9,33]. Significant diversity in seed colors was observed, with darker seeds exhibiting higher levels of active compounds. Groups with darker seeds (e.g., Group 1) had higher anthocyanin content and antioxidant capacities, whereas these parameters were the lowest in groups with lighter seeds (e.g., Group 2). Groups with darker seeds (Groups 1 and 10) displayed the highest anthocyanin, phenolic, and flavonoid contents as well as antioxidant activities. A positive correlation was noted between darker seed colors and antioxidant activities, whereas lighter seed colors were negatively correlated with antioxidant activities. These findings support the potential of CW mutants as valuable breeding materials for biofortification and the development of nutritionally superior wheat varieties. Incorporating these traits into wheat breeding programs can lead to the development of wheat varieties with enhanced nutritional value, playing a critical role in addressing global malnutrition. Antioxidant-rich wheat varieties, in particular, could be highly beneficial for populations with limited access to diverse diets, contributing to improved public health outcomes worldwide. Despite the strong correlation between seed color and functional traits, it is important to acknowledge that other factors may also influence antioxidant properties. For instance, environmental conditions and secondary metabolites other than the measured parameters may contribute to the observed variations. Pigmentation in wheat seeds is often regulated by key genes, such as *TaMYB* and *bHLH* transcription factors, which can not only control anthocyanin biosynthesis but also potentially influence pathways involved in the synthesis of other bioactive compounds [17,34]. Future studies integrating genomic tools and transcriptomic analysis should further elucidate these genetic pathways, offering insights into the coregulation of pigmentation and bioactive compound accumulation.

Notably, despite the apparent correlation between seed color and phytochemical content, gamma irradiation, which induced these color variations, also led to significant diversity in agronomic traits among the mutants. Although the mutants within each group exhibited similar seed colors based on L*, a*, and b* values, they displayed considerable variation in other traits, such as HD, PH, SL, spike morphology (awn presence), and TSW. This finding highlights the complex nature of mutagenesis, wherein similar phenotypic characteristics, such as seed color, may arise from different underlying genetic changes, resulting in distinct agronomic phenotypes. For instance, Groups 1 and 10, classified based on seed color, exhibited vast differences in PH, SL, and other agronomic traits despite their similar color. They also displayed significant variation in PH, with some mutants being tall and others relatively short. Similarly, the presence of awns, a key agronomic trait, varied within the groups. These variations suggest that although seed color can be used as a phenotypic marker for bioactive compounds, it may not be a reliable indicator of agronomic uniformity in mutant populations. In this study, darker seed mutants, such as those in Group 1, exhibited higher anthocyanin content and antioxidant activities, which are desirable functional traits. Occasionally, these benefits were offset by agronomic trade-offs, such as reduced uniformity in PH or lower TSW, which are crucial for large-scale cultivation. Conversely, lighter seed mutants, such as those in Group 2, demonstrated greater agronomic stability but lacked the functional advantages observed in darker seed mutants. These findings highlight the need to balance functional and agronomic traits in breeding programs and emphasize the importance of integrating phenotypic and genetic approaches to minimize trade-offs. The agronomic traits measured in this study may not directly correlate with seed color, antioxidant levels, or the content of other bioactive compounds, because the data were collected during a single growing season under specific field conditions. Nevertheless, the significant diversity observed in traits such as PH, flowering time, and spike morphology among mutant lines with similar seed colors underscores the potential of gamma irradiation to generate phenotypic variability beyond that achievable through conventional breeding. Although agronomic traits may be influenced by environmental factors and require validation across multiple seasons, these findings highlight the value of mutation breeding in developing wheat lines with diverse agronomic characteristics, contributing to both yield-related traits and functional properties such as antioxidant activity and bioactive compound content.

Gamma irradiation is effective in producing a broad spectrum of seed colors; it induces genetic mutations that impact various agronomic traits. This variability is indicative of the randomness inherent to the process of mutagenesis, where changes to one trait, such as seed pigmentation, may be linked to other changes in the genome. Despite its randomness, gamma irradiation offers unique opportunities for breeding programs by introducing novel trait combinations. The observed phenotypic diversity allows for the selection of lines with desirable functional and agronomic properties; however, this diversity may complicate large-scale breeding efforts, as extensive evaluation and selection are required to balance functional traits, such as anthocyanin content, with agronomic traits such as SL and PH stability. For instance, some lighter seed color mutants, despite reduced anthocyanin content, showed stable SL and PH values, suggesting potential utility in breeding programs targeting yield stability or adaptation to specific environments. However, these advantages need to be weighed against the complexity of maintaining uniformity in large-scale cultivation. By combining phenotypic markers, such as seed color, with advanced analytical tools, such as *K*-means clustering and PCA, this study highlights how mutation breeding can be used to evaluate complex trait correlations. Although this study did not incorporate genomic tools, future research should integrate approaches such as genome-wide association studies and quantitative trait locus mapping. These techniques may offer deeper insights into the genetic basis of numerous traits, including seed color, antioxidant activities, and agronomic performance. Balancing functional traits with critical agronomic characteristics remains a key challenge but also represents a significant opportunity for advancing mutation breeding efforts.

This study provides novel insights into the relationship between seed color variation and antioxidant properties in colored wheat mutants generated through gamma irradiation. Unlike previous studies that focused on naturally occurring colored wheat varieties, our approach induced a wide spectrum of seed coat colors through targeted gamma irradiation, expanding the available genetic diversity. Additionally, the application of *K*-means clustering for the classification of mutant lines based on seed color parameters (L*, a*, b*) is a key methodological advancement, allowing for a more systematic and data-driven selection process. The consistency observed between *K*-means clustering and PCA results further reinforces the robustness of seed color as a potential phenotypic marker for functional traits. These contributions distinguish this study from previous research and provide a valuable framework for integrating mutation breeding with phenotypic and biochemical assessments in wheat improvement programs.

These results provide a valuable foundation for future efforts to balance functional traits, such as antioxidant properties, with desirable agronomic characteristics, ultimately contributing to the development of nutritionally enhanced and agronomically robust wheat varieties. This study focused on differences in antioxidant activity based on seed color in CW mutant lines. However, future research should include protein and gluten content analysis along with baking trials to provide a more comprehensive understanding of the quality and functionality of these wheat varieties. In this regard, gamma irradiation offers a robust tool for generating genetic diversity, allowing for the development of wheat varieties with enhanced functional properties, although it also presents challenges in terms of maintaining consistency in agronomic traits. Overall, this study demonstrates that integrating phenotypic, biochemical, and agronomic assessments through statistical clustering and multivariate analyses provides a comprehensive framework for harnessing mutagenesis in crop improvement.

## 5. Conclusions

This study highlights the potential of gamma-radiation-induced mutagenesis to diversify both functional and agronomic traits in CW. The consistency between the results of *K*-means clustering and PCA demonstrates a strong correlation between seed color and antioxidant activity, with darker mutants exhibiting higher levels of anthocyanins and antioxidant activities. These findings have practical applications in developing wheat varieties with enhanced functional traits, which can be used in functional food products aimed at improving health outcomes, such as reducing oxidative stress and preventing chronic diseases. However, the variability in agronomic traits observed among the mutants suggests the need to balance functional traits with critical agronomic characteristics for large-scale cultivation. Future research should validate these traits under diverse environmental conditions, including multilocation and multiseason trials, to ensure trait stability. Conducting experiments under stress conditions, such as drought, low temperatures, or salinity, would provide additional insights into how environmental factors influence the accumulation of bioactive compounds. Considering that antioxidant-related compounds are often produced in response to environmental stress, such trials will help confirm the practical relevance of these findings across multiple agricultural systems.

## Figures and Tables

**Table 1:** Classification of colored wheat mutants based on L*, a*, and b* values.

Group	Number	L*	a*	b*
1	67	25.04 ± 2.45 ^a^	5.48 ± 1.13 ^d^	5.60 ± 1.20 ^a^
2	39	46.11 ± 2.94 ^j^	7.56 ± 0.95 ^g^	23.16 ± 1.97 ^j^
3	189	26.97 ± 1.87 ^b^	4.93 ± 0.91 ^a^	6.59 ± 0.88 ^b^
4	183	28.10 ± 2.79 ^c^	5.23 ± 1.03 ^ab^	7.82 ± 0.93 ^c^
5	56	34.19 ± 4.60 ^g^	6.21 ± 1.00 ^h^	13.29 ± 1.30 ^g^
6	97	41.97 ± 3.72 ^h^	6.04 ± 0.96 ^f^	17.02 ± 1.25 ^h^
7	84	31.34 ± 2.82 ^f^	6.19 ± 1.26 ^fg^	11.54 ± 1.13 ^f^
8	109	29.93 ± 2.45 ^e^	5.81 ± 1.10 ^e^	10.12 ± 1.04 ^e^
9	85	43.51 ± 4.01 ^i^	6.77 ± 0.95 ^i^	19.64 ± 1.60 ^i^
10	160	29.02 ± 2.42 ^d^	5.35 ± 1.05 ^j^	8.79 ± 0.91 ^d^
Total	1069	33.62 ± 6.88	5.96 ± 1.21	13.36 ± 4.89

Superscript letters (a–j) indicate significant differences among the means based on Duncan’s multiple range test (*p* < 0.05).

**Table 2:** Diversity and individual effects of seed color across groups on the active compounds and antioxidant activities in colored wheat mutants.

Parameter	Value	Control		Group										
		CW	K	1	2	3	4	5	6	7	8	9	10	Total (Except Control)
Anthocyanins (units)	Min.	4.713	3.414	4.914	2.946	4.046	3.614	3.280	3.147	3.880	3.579	2.979	4.180	2.946
	Max.	4.948	3.581	6.781	3.280	4.881	10.082	4.781	3.615	6.147	6.515	3.481	6.414	10.082
	Mean	4.813	3.514	5.6195 ^f^	3.147 ^a^	4.578 ^d^	5.414 ^c^	3.747 ^abc^	3.367 ^ab^	5.133 ^ef^	4.736 ^de^	3.275 ^ab^	5.375 ^f^	4.439
	SD	0.108	0.079	0.650	0.108	0.226	2.758	0.558	0.157	0.845	1.068	0.136	0.844	1.368
	CV	2.2	2.2	11.6	3.4	4.9	50.9	14.9	4.7	16.4	22.6	4.2	15.7	30.8
Proanthocyanidins (μg/mL)	Min.	5.393	4.585	4.384	4.283	4.585	4.484	4.384	4.283	4.282	4.080	4.182	4.384	4.080
	Max.	6.606	5.595	8.929	5.293	4.989	5.798	8.828	4.990	6.808	7.515	6.404	5.394	8.929
	Mean	5.898	4.989	5.242	4.586	4.729	5.040	5.250	4.687	5.032	5.074	4.973	4.721	4.933
	SD	0.564	0.478	1.206	0.286	0.125	0.471	1.232	0.247	0.770	0.939	0.569	0.280	0.731
	CV	9.5	9.5	23.0	6.2	2.6	9.3	23.4	5.3	15.3	18.5	11.4	5.9	14.8
Phenolic compounds (nmole)	Min.	1.519	1.370	1.465	1.248	1.425	1.303	1.289	1.316	1.303	1.357	1.329	1.669	1.248
	Max.	1.818	1.546	2.049	1.533	1.859	2.062	1.777	1.655	2.185	1.858	1.547	2.049	2.184
	Mean	1.650	1.438	1.708 ^de^	1.378 ^a^	1.559 ^bcd^	1.588 ^cd^	1.459 ^abc^	1.489 ^abc^	1.768 ^e^	1.537 ^abc^	1.424 ^ab^	1.786 ^e^	1.570
	SD	0.136	0.084	0.202	0.086	0.120	0.291	0.148	0.098	0.303	0.147	0.064	0.122	0.218
	CV	8.2	5.8	11.8	6.2	7,7	18.3.	10.1	6.6	17.1	9.6	4.5	6.8	13.9
Flavonoids (μg/g)	Min.	63.80	60.66	63.17	57.53	60.04	61.92	57.53	63.17	62.55	61.92	60.04	63.80	57.53
	Max.	70.07	65.68	78.23	65.68	75.09	74.47	77.60	76.35	75.72	71.96	70.08	72.58	78.23
	Mean	67.25	63.64	73.10 ^d^	61.50 ^a^	68.66 ^bc^	67.82 ^bc^	65.58 ^b^	69.97 ^bcd^	69.24 ^bc^	66.57 ^b^	65.53 ^b^	68.45 ^bc^	67.64
	SD	3.05	1.97	4.88	2.86	5.04	3.96	6.45	4.09	3.90	2.91	3.43	2.96	5.00
	CV	4.5	3.0	6.7	4.7	7.3	5.8	9.8	5.8	5.6	4.4	5.2	4.3	7.4
ABTS (%)	Min.	22.014	18.220	21.679	13.653	17.517	21.311	12.292	17.236	20.295	17.580	10.382	17.581	10.382
	Max.	22.716	21.311	30.304	21.402	26.937	27.277	21.171	25.527	32.125	27.906	22.509	29.566	32.125
	Mean	22.295	19.718	24.974 ^cd^	17.598 ^a^	22.057 ^bc^	24.692 ^cd^	17.706 ^a^	19.584 ^ab^	25.179 ^d^	21.977 ^bc^	17.527 ^a^	23.032 ^c^	21.433
	SD	0.332	1.384	3.314	2.063	2.719	2.270	3.104	2.803	3.907	3.589	4.241	4.606	4.388
	CV	1.4	7.0	13.3	11.7	12.3	9.2	17.5	14.3	15.5	16.3	24.2	20.0	20.5
DPPH (%)	Min.	24.931	23.299	20.533	10.464	19.946	20.350	8.696	12.062	20.730	15.499	6.689	24.384	6.689
	Max.	27.759	27.443	36.927	21.260	33.596	30.952	28.942	31.043	43.812	34.327	29.583	43.813	34.847
	Mean	26.755	24.742	31.659 ^c^	15.537 ^a^	26.931 ^cde^	25.549 ^cd^	19.325 ^ab^	22.053 ^bc^	29.874 ^de^	26.136 ^cd^	20.296 ^ab^	30.067 ^e^	24.743
	SD	1.415	2.107	5.287	3.383	3.479	3.335	6.978	6.709	8.972	6.148	8.284	3.592	7.631
	CV	5.2	8.5	16.7	21.8	12.9	13.1	36.1	30.4	30.0	23.5	40.8	11.9	30.8
FRAP (%)	Min.	5.093	4.172	4.492	3.628	4.079	4.285	3.6279	4.210	4.135	4.079	3.609	4.248	3.609
	Max.	5.300	4.229	6.541	4.756	5.244	5.094	5.075	5.376	5.582	5.827	5.150	6.2789	6.5418
	Mean	5.194	4.204	5.133 ^d^	4.180 ^a^	4.641 ^bc^	4.743 ^bcd^	4.212 ^a^	4.680 ^bc^	4.823 ^bcd^	4.795 ^bcd^	4.470 ^ab^	5.009 ^ab^	4.669
	SD	0.092	0.025	0.632	0.383	0.379	0.279	0.484	0.385	0.458	0.553	0.476	0.675	0.551
	CV	1.7	0.6	12.3	9.2	8.2	5.9	11.5	8.2	9.5	11.5	10.6	13.5	11.8
TAC (nmole)	Min.	3.881	3.247	3.543	3.135	3.266	3.373	3.141	3.329	3.511	3.348	3.298	3.436	3.134
	Max.	4.075	3.561	4.383	3.781	3.982	3.994	3.612	4.000	4.483	4.145	3.925	4.132	4.483
	Mean	4.002	3.408	3.893 ^de^	3.427 ^ab^	3.622 ^c^	3.641 ^c^	3.388 ^a^	3.678 ^c^	3.935 ^e^	3.687 ^c^	3.598 ^b^	3.719 ^cd^	3.659
	SD	0.094	0.140	0.234	0.221	0.185	0.194	0.134	0.208	0.315	0.221	0.191	0.230	0.266
	CV	2.3	4.1	6.0	6.4	5.1	5.3	4.0	5.7	8.0	6.0	5.3	6.2	7.3

CV: coefficient of variation. Superscript letters (a–f) indicate significant differences among the means based on Duncan’s multiple range test (*p* < 0.05).

**Table 3:** Agronomic traits of colored wheat mutants and wild-type wheat evaluated during 2022–2023.

Group	Line	Plant Height	Spike Length	Days to Heading	TSW	NKS
1	650	88.0 ± 0.9	8.0 ± 0.3	191	37.6 ± 0.6	50.0 ± 2.7
	656	83.3 ± 1.4	7.7 ± 0.3	192	39.6 ± 0.2	66.3 ± 6.1
	798	114.3 ± 3.6	13.5 ± 1.3	202	39.8 ± 2.1	60.3 ± 9.8
	871	115 ± 7.9	7.3 ± 0.2	197	44.8 ± 2.1	42.7 ± 4.2
2	383	104.3 ± 5.4	12.6 ± 0.5	196	50.7 ± 1.4	53. 7 ± 4.0
	995	111.3 ± 3.1	8.1 ± 0.2	198	35.2 ± 0.9	40.3 ± 3.1
	1007	114.0 ± 3.2	11.5 ± 0.4	199	35.7 ± 0.7	49.0 ± 2.4
	1030	118.0 ± 3.2	10.9 ± 0.2	200	46.4 ± 1.6	53.3 ± 2.7
3	627	67.3 ± 1.9	7.5 ± 0.3	191	42.1 ± 2.3	48. 7 ± 6.9
	841	111.3 ± 1.0	6.1 ± 0.2	188	47.9 ± 1.2	41.0 ± 2.7
	625	77.7 ± 2.3	7.0 ± 0.2	190	39.5 ± 1.7	46.0 ± 3.2
	925	108.7 ± 1.9	7.3 ± 0.2	191	48.3 ± 1.3	46.0 ± 1.5
4	803	109. 7 ± 1.4	8.0 ± 0.2	194	43.9 ± 1.9	44.0 ± 5.7
	854	84.7 ± 2.3	6.5 ± 0.1	190	35.8 ± 2.4	42.0 ± 3.9
	1036	85.3 ± 2.7	7.1 ± 0.5	190	41.9 ± 1.6	52.3 ± 5.1
	1122	73.3 ± 3.1	6.8 ± 0.5	194	32.2 ± 1.8	47 ± 4.1
5	1144	119.3 ± 3.4	6.6 ± 0.3	188	43.5 ± 3.0	39. 7 ± 3.6
	431	130 ± 3.9	9.3 ± 0.4	187	48.2 ± 2.2	45.7 ± 1.4
	153	84.7 ± 3.47	9.1 ± 0.2	187	38.3 ± 1.3	43. 7 ± 3.6
	366	125 ± 2.7	8.3 ± 0.4	200	30.5 ± 1.1	38.3 ± 2.7
6	1079	40.3 ± 1.4	6.3 ± 0.5	197	24.9 ± 0.3	49.3 ± 5.8
	291	133.0 ± 4.7	9.8 ± 0.7	201	46.7 ± 2.5	31.3 ± 2.9
	508	127.3 ± 2.7	11.0 ± 0.4	199	42.3 ± 1.4	44. 7 ± 5.4
	221	32. 7 ± 2.3	8.3 ± 0.4	197	33.3 ± 1.0	47. 7 ± 6.3
7	1021	120. 7 ± 1.9	7.3 ± 0.8	194	40.9 ± 1.6	42.0 ± 4.5
	483	105.7 ± 2.7	7.2 ± 0.2	192	37.4 ± 0.2	47.3 ± 0.5
	1019	140.0 ± 1.8	9.93 ± 0.3	192	47.3 ± 2.1	50.0 ± 4.1
	885	123.0 ± 1.8	6.6 ± 0.4	189	43.9 ± 1.7	33.0 ± 4.7
8	376	105.7 ± 2.7	6.0 ± 0.2	189	44.4 ± 0.2	54.0 ± 4.1
	1017	101.7 ± 3.1	11.6 ± 1.1	198	50.1 ± 1.2	42.7 ± 1.4
	983	95.3 ± 2.3	6.9 ± 0.2	191	40.1 ± 2.1	40.7 ± 3.4
	42	114.7 ± 2.3	6.6 ± 0.2	192	40.9 ± 0.9	52.0 ± 2.4
9	1209	107.2 ± 1.8	6.6 ± 0.1	192	42.9 ± 1.2	61.3 ± 3.6
	573	130.0 ± 3.9	8.6 ± 0.5	190	38.3 ± 2.9	33.0 ± 0.9
	12	116.3 ± 3.4	7.5 ± 0.1	194	39.4 ± 1.7	33.7 ± 2.9
	453	103.7 ± 3.4	6.7 ± 0.3	191	36.3 ± 1.2	48.7 ± 2.7
10	367	67.3 ± 3.4	9.2 ± 0.3	196	34.8 ± 1.3	68.7 ± 3.1
	192	103.7 ± 2.7	7.8 ± 0.2	192	37.2 ± 0.8	50.0 ± 2.4
	635	76.3 ± 3.4	8.0 ± 0.1	191	41.0 ± 0.4	52.7 ± 6.5
	944	110.0 ± 2.7	6.7 ± 0.4	197	41.1 ± 1.4	56.7 ± 4.4
Wild type	CW	75.3 ± 2.7	7.1 ± 0.2	191	42.3 ± 2.4	59.0 ± 4.1
	K	87.2 ± 3.9	7.9 ± 0.4	178	46.0 ± 2.5	53.7 ± 3.6

## Data Availability

The original contributions presented in the study are included in the article/Supplementary Material, further inquiries can be directed to the corresponding author.

## Supplementary Materials

The following supporting information can be downloaded at: https://www.mdpi.com/article/10.3390/foods14030487/s1, Table S1: Hunter *L**, *a**, and *b** color diagram for seed color in 1069 mutant lines of colored wheat.

## Abbreviations

The following abbreviations are used in this manuscript:

ABTS	2,2′-Azino-bis (3-ethylbenzothiazoline-6-sulfonic acid) diammonium salt
DPPH	2,2-Diphenyl-1-picrylhydrazyl
FRAP	Ferric reducing antioxidant power
HD	Days to heading
NKS	Number of kernels per spike
PCA	Principal component analysis
PH	Plant height
SL	Spike length
TAC	Total antioxidant activity
TSW	Thousand-seed weight

## References

[1] Balfourier F., Bouchet S., Robert S., De Oliveira R., Rimbert H., Kitt J., Choulet F., Paux E., International Wheat Genome Sequencing Consortium, Breed Wheat Consortium (2019). Worldwide phylogeography and history of wheat genetic diversity. Sci. Adv..

[2] Shewry P.R., Hey S.J. (2015). The contribution of wheat to human diet and health. Food Energy Secur..

[3] Sharma S., Chunduri V., Kumar A., Kumar R., Khare P., Kondepudi K.K., Bishnoi M., Garg M. (2018). Anthocyanin bio-fortified colored wheat: Nutritional and functional characterization. PLoS ONE.

[4] Hosseinian F.S., Li W., Beta T. (2008). Measurement of anthocyanins and other phytochemicals in purple wheat. Food Chem..

[5] Fan X., Xu Z., Wang F., Feng B., Zhou Q., Cao J., Ji G., Yu Q., Liu X., Liao S. (2020). Identification of colored wheat genotypes with suitable quality and yield traits in response to low nitrogen input. PLoS ONE.

[6] Razgonova M.P., Zakharenko A.M., Gordeeva E.I., Shoeva O.Y., Antonova E.V., Pikula K.S., Koval L.A., Khlestkina E.K., Golokhvast K.S. (2021). Phytochemical analysis of phenolics, sterols, and terpenes in colored wheat grains by liquid chromatography with tandem mass spectrometry. Molecules.

[7] Kaur S., Kumari A., Sharma N., Pandey A.K., Garg M. (2022). Physiological and molecular response of colored wheat seedlings against phosphate deficiency is linked to accumulation of distinct anthocyanins. Plant Physiol. Biochem..

[8] Abdel-Aal E.M., Young J.C., Rabalski I. (2006). Anthocyanin composition in black, blue, pink, purple, and red cereal grains. J. Agric. Food Chem..

[9] Liu Q., Qiu Y., Beta T. (2010). Comparison of antioxidant activities of different colored wheat grains and analysis of phenolic compounds. J. Agric. Food Chem..

[10] Garg M., Kaur S., Sharma A., Kumari A., Tiwari V., Sharma S., Kapoor P., Sheoran B., Goyal A., Krishania M. (2022). Rising demand for healthy foods-anthocyanin biofortified colored wheat is a new research trend. Front. Nutr..

[11] Garg M., Chawla M., Chunduri V., Kumar R., Sharma S., Sharma N.K., Kaur N., Kumar A., Mundey J.K., Saini M.K. (2016). Transfer of grain colors to elite wheat cultivars and their characterization. J. Cereal Sci..

[12] Ficco D.B., Mastrangelo A.M., Trono D., Borrelli G.M., De Vita P., Fares C., Beleggia R., Platani C., Papa R. (2014). The colours of durum wheat: A review. Crop Pasture Sci..

[13] Zeven A.C. (1991). Wheats with Purple and Blue Grains: A Review. Euphytica.

[14] Abdel-Aal E.M., Choo T., Dhillon S., Rabalski I. (2012). Free and bound phenolic acids and total phenolics in black, blue, and yellow barley and their contribution to free radical scavenging capacity. Cereal Chem..

[15] Putta S., Yarla N.S., Kumar K.E., Lakkappa D.B., Kamal M.A., Scotti L., Scotti M.T., Ashraf G.M., Rao B.S.B., Reddy G.V. (2018). Preventive and therapeutic potentials of anthocyanins in diabetes and associated complications. Curr. Med. Chem..

[16] Tian S., Chen Z., Wei Y. (2018). Measurement of colour-grained wheat nutrient compounds and the application of combination technology in dough. J. Cereal Sci..

[17] Li L., Zhang H., Liu J., Huang T., Zhang X., Xie H., Guo Y., Wang Q., Zhang P., Qin P. (2023). Grain color formation and analysis of correlated genes by metabolome and transcriptome in different wheat lines at maturity. Front. Nutr..

[18] Du Y., Feng Z., Wang J., Jin W., Wang Z., Guo T., Chen Y., Feng H., Yu L., Li W. (2022). Frequency and spectrum of mutations induced by gamma rays revealed by phenotype screening and whole-genome re-sequencing in *Arabidopsis thaliana*. Int. J. Mol. Sci..

[19] Hase Y., Satoh K., Seito H., Oono Y. (2020). Genetic consequences of acute/chronic gamma and carbon ion irradiation of *Arabidopsis thaliana*. Front. Plant Sci..

[20] Rutger J.N., Peterson M.L., Hu C.H. (1977). Registration of Calrose 76 rice 1 (Reg. no. 45). Crop Sci..

[21] Jamil M. (2002). Study of genetic variation in yield components of wheat cultivar bukhtwar-92 as induced by γ radiation. Asian J. Plant Sci..

[22] Cheng X., Chai L., Chen Z., Xu L., Zhai H., Zhao A., Peng H., Yao Y., You M., Sun Q. (2015). Identification and characterization of a high kernel weight mutant induced by gamma radiation in wheat (*Triticum aestivum* L.). BMC Genet..

[23] Zulfiqar S., Ishfaq S., Ikram M., Nawaz M.A., Rahman M. (2021). Characterization of gamma-rays-induced spring wheat mutants for morphological and quality traits through multivariate and gt bi-plot analysis. Agronomy.

[24] Howe E.A., Sinha R., Schlauch D., Quackenbush J. (2011). RNA-seq analysis in MeV. Bioinformatics.

[25] Mita S., Murano N., Akaike M., Nakamura K. (1997). Mutants of *Arabidopsis thaliana* with pleiotropic effects on the expression of the gene for *β*-amylase and on the accumulation of anthocyanin that are inducible by sugars. Plant J..

[26] Brand-Williams W., Cuvelier M.E., Berset C. (1995). Use of a free radical method to evaluate antioxidant activity. LWT-Food Sci. Technol..

[27] Re R., Pellegrini N., Proteggente A., Pannala A., Yang M., Rice-Evans C. (1999). Antioxidant activity applying an improved abts radical cation decolorization assay. Free Radic. Biol. Med..

[28] Kohyama N., Chono M., Nakagawa H., Matsuo Y., Ono H., Matsunaka H. (2017). Flavonoid compounds related to seed coat color of wheat. Biosci. Biotechnol. Biochem..

[29] Khalid A., Hameed A., Tahir M.F. (2023). Wheat quality: A review on chemical composition, nutritional attributes, grain anatomy, types, classification, and function of seed storage proteins in bread making quality. Front. Nutr..

[30] Gamel T.H., Saeed S.M.G., Ali R., Abdel-Aal E.M. (2023). Purple wheat: Food development, anthocyanin stability, and potential health benefits. Foods.

[31] Sharma A., Yadav M., Tiwari A., Ali U., Krishania M., Bala M., Mridula D., Sharma P., Goudar G., Roy J.K. (2023). A comparative study of colored wheat lines across laboratories for validation of their phytochemicals and antioxidant activity. J. Cereal Sci..

[32] Sytar O., Bośko P., Živčák M., Brestic M., Smetanska I. (2018). Bioactive phytochemicals and antioxidant properties of the grains and sprouts of colored wheat genotypes. Molecules.

[33] Hong M.J., Kim D.Y., Nam B.M., Ahn J., Kwon S., Seo Y.W., Kim J. (2019). characterization of novel mutants of hexaploid wheat (*Triticum aestivum* L.) with various depths of purple grain color and antioxidant capacity. J. Sci. Food Agric..

[34] Flores P.C., Yoo J.S., Kim D.Y., Seo Y.W. (2022). Transcriptome analysis of myb genes and patterns of anthocyanin accumulation during seed development in wheat. Evol. Bioinform. Online.

